# Organic–inorganic nanosystems

**DOI:** 10.3762/bjnano.2.41

**Published:** 2011-07-12

**Authors:** Paul Ziemann

**Affiliations:** 1 Institute of Solid State Physics, Ulm University, 89081 Ulm, Germany

A natural goal of most nanoscience projects, independent of the specific subfield they belong to, is to create a system providing novel functions which originate from the properties of its constituent nanoscale parts. Although the preparation of individual nanoobjects such as dots or wires of various materials is in principle possible, the analysis of their chemical and physical properties still poses a serious experimental challenge. Thus, to arrive at a certain minimum level of signal strength for a given analysis technique, in most cases ensembles of nanoobjects are needed. However, this immediately results in the requirement for a narrow size distribution of the nanoobjects, else their desired new size dependent properties will be completely smeared out. Additional conditions such as control of the chemical composition of the structures or of their spatial arrangement add further difficulties to the preparation. Finally, excluding time consuming, and thus costly, sequential top-down methods, one is almost naturally led to self-organizing bottom-up processes.

At this point organic**–**inorganic nanosystems enter the stage. Imagine an organic moiety such as a diblock-copolymer, in an appropriate solvent, forming an even more complex building block such as a nanoscaled micelle, which, in turn, can be deposited onto an inorganic substrate such as a Si-wafer by dip coating. Due to the presence of a complex hierarchy of interactions, such as those between copolymers, or copolymer**–**solvent, solvent**–**substrate or copolymer**–**substrate interactions, a rich spectrum of resulting micellar arrangements on top of a given inorganic substrate will emerge. This variety can be even further extended by the application to pre-patterned substrates acting as templates.

In the above example, diblock-copolymers will form spherical micelles within a certain window of block lengths in a properly chosen solvent, and such micelles can be used as carriers for a metal precursor during their self-organization on top of a substrate. This specific case is immediately generalized to the idea of depositing molecular, macromolecular or supramolecular organic species on top of inorganic platforms. By exploiting molecular self-organization, one finally arrives at a functional system with novel properties.

Much progress has been made during the last decade based on this idea of exploiting the self-organization of organic building blocks on top of inorganic supports, and a huge number of self-assembled structures have been prepared. A direct application of this approach is to use such assemblies as templates or masks for subsequent further deposition or etching steps. Applied in this way, the method is a direct extension of the seminal work by Fischer and Zingsheim on hexagonal ordered arrays of polystyrene colloids serving as masks for subsequent metal evaporations [1].

In other cases, based on precursor loaded micelles or, more generally, colloids, the organic carriers are completely removed after their self-organization by various plasma treatments while the precursor compounds are transformed into metal oxides or, finally, into metals. In this way, hexagonal ordered arrays of metal nanoparticles can be obtained with controllable particle size and inter-particle distance. In that case, the organic**–**inorganic nanosystem simply serves as an intermediate step towards the sought after particle arrangement. An application of this technique for the preparation of magnetic nanoparticles is described in another recent Thematic Series about the preparation, properties and applications of magnetic nanoparticles in the *Beilstein Journal of Nanotechnology* [2].

In the present context, the wide field of ligand-stabilized nanoparticles should also be mentioned. Here, each particle represents an organic**–**inorganic system, opening up almost unlimited possibilities for the chemical functionalization of the particles and, hence, making them into selective docking stations for other molecules. Exploiting this selectivity, various chemical sensors have been developed with specific applications in biology and medicine. Closely related to this topic are drug delivery “absorption-active” nanoparticles, which are again surface functionalized organic**–**inorganic systems that can be specifically targeted towards tumor cells.

Up to this point, the organic part of the considered hybrids provided self-organization and specificity for subsequent chemical reactions or, in the simplest case, served as a spacer to avoid aggregation in a system of corresponding nanoobjects. However, there is yet another highly active field where the organic component delivers the sought after functionality of a device: Organic electronics. Here, doped π-conjugated oligomers and polymers play an important role due to their semiconducting behavior. As in standard electronics, the combination of p- and n-doped organic components leads to device applications such as organic light emitting diodes (OLED), organic field effect transistors (OFET) and organic solar cells, to name but a few. In all cases, the organic component must be brought into contact with a conducting electrode, usually a metal or a highly doped semiconductor. Each of these contacts represents an organic**–**inorganic interface with gradients on the nanoscale. Due to the importance of these interfaces on the transport properties of the devices, the electronic properties of various arrangements of organic molecules on top of metals must be studied. For the analysis of single molecules, the most promising technique is Scanning Tunneling Microscopy (STM) and its spectroscopic variant (STS), while for larger coverages standard photoelectron spectroscopies such UPS or XPS **–** often synchrotron based **–** also play an important role.

This broad spectrum of preparational approaches, analytical tools and sought after functionalities, all related to organic**–**inorganic nanosystems, are addressed in the present thematic series. The topic naturally demands an interdisciplinary cooperation between chemists and physicists, experimentalists and theoreticians, and this is reflected in the statistics of the authors contributing to this series. Practically all contributions have been at least in part funded by the German Science Foundation (DFG), many of them within the Collaborative Research Center (SFB) 569 dealing with the *“Hierarchical Structure Formation and Function of Organic***–***Inorganic Nanosystems”*. Thus, in addition to acknowledging the contributions of all authors and their teams, I would especially like to thank DFG for generous long term support. Special thanks also go to Barbara Jörg, Ursula von Gliscynski, and Frank-Dieter Kuchta for providing us with their advice over many years.

Paul Ziemann

Ulm, July 2011
